# Low- and High-Drag Intermittencies in Turbulent Channel Flows

**DOI:** 10.3390/e22101126

**Published:** 2020-10-04

**Authors:** Rishav Agrawal, Henry C.-H. Ng, Ethan A. Davis, Jae Sung Park, Michael D. Graham, David J.C. Dennis, Robert J. Poole

**Affiliations:** 1 School of Engineering, University of Liverpool, Liverpool L69 3GH, UK; ad5289@coventry.ac.uk (R.A.); hchng@liverpool.ac.uk (H.C.-H.N.); David.Dennis@liverpool.ac.uk (D.J.C.D.); 2 Fluid and Complex Systems Research Centre, Coventry University, Coventry CV1 5FB, UK; 3 Department of Mechanical and Materials Engineering, University of Nebraska-Lincoln, Lincoln, NE 68588-0526, USA; ethan.davis@huskers.unl.edu (E.A.D.); jaesung.park@unl.edu (J.S.P.); 4 Department of Chemical and Biological Engineering, University of Wisconsin-Madison, Madison, WI 53706, USA; mdgraham@wisc.edu

**Keywords:** hibernating turbulence, hot-film anemometry, turbulence, channel flow

## Abstract

Recent direct numerical simulations (DNS) and experiments in turbulent channel flow have found intermittent low- and high-drag events in Newtonian fluid flows, at $Re_{\tau }=u_{\tau }h/\nu$ between 70 and 100, where $u_{\tau }$, *h* and ν are the friction velocity, channel half-height and kinematic viscosity, respectively. These intervals of low-drag and high-drag have been termed “hibernating” and “hyperactive”, respectively, and in this paper, a further investigation of these intermittent events is conducted using experimental and numerical techniques. For experiments, simultaneous measurements of wall shear stress and velocity are carried out in a channel flow facility using hot-film anemometry (HFA) and laser Doppler velocimetry (LDV), respectively, for $Re_{\tau }$ between 70 and 250. For numerical simulations, DNS of a channel flow is performed in an extended domain at $Re_{\tau }$ = 70 and 85. These intermittent events are selected by carrying out conditional sampling of the wall shear stress data based on a combined threshold magnitude and time-duration criteria. The use of three different scalings (so-called outer, inner and mixed) for the time-duration criterion for the conditional events is explored. It is found that if the time-duration criterion is kept constant in inner units, the frequency of occurrence of these conditional events remain insensitive to Reynolds number. There exists an exponential distribution of frequency of occurrence of the conditional events with respect to their duration, implying a potentially memoryless process. An explanation for the presence of a spike (or dip) in the ensemble-averaged wall shear stress data before and after the low-drag (or high-drag) events is investigated. During the low-drag events, the conditionally-averaged streamwise velocities get closer to Virk’s maximum drag reduction (MDR) asymptote, near the wall, for all Reynolds numbers studied. Reynolds shear stress (RSS) characteristics during these conditional events are investigated for $Re_{\tau }$ = 70 and 85. Except very close to the wall, the conditionally-averaged RSS is higher than the time-averaged value during the low-drag events.

## 1. Introduction

In the past few decades, the understanding of near-wall coherent structures has been greatly improved via the discovery of travelling-wave (TW) solutions [1]. These TW solutions were first obtained by Nagata [2] for plane Couette flow. They are non-trivial invariant solutions to the Navier–Stokes equation and are also sometimes called “exact coherent states (ECS)”. Later, Waleffe [3,4] found ECS solutions for plane channel flow. The spatial structure of these solutions is similar to the commonly observed structure of near-wall turbulence: mean flow with counter-rotating streamwise vortices and alternating low- and high-speed streaks. Most of these ECS solutions are observed to occur in pairs at a saddle-node bifurcation point, arising at a finite value of Reynolds number. The upper branch solution has a higher fluctuation amplitude and higher drag than the lower branch solution [2,3,4,5].

One way to investigate the complex turbulent dynamics using TW solutions is to employ “minimal flow units”. The minimal flow units or MFU denotes the smallest computational domain where turbulence can persist [6] at a given Reynolds number. Jiménez and Moin [6] observed a cyclic and intermittent behaviour of the fluctuations of all important quantities while employing MFU to study plane channel flow. They also observed a rapid increase in the fluctuations and wall shear stress during the “active” part of the cycle. Later, Hamilton et al. [7] and Jiménez and Pinelli [8] further studied this cycle and observed that during the time when the wall shear stress is near its lowest values the streamwise variation of the flow is also reduced. The presence of intermittency in Newtonian turbulent flow has also been investigated earlier by McComb [9]. Xi and Graham [10] carried out DNS in an MFU for low Reynolds number, $Re_{\tau }=u_{\tau }h/\nu =85$ for both Newtonian and viscoelastic flows. Here, $u_{\tau }$, *h* and ν are the friction velocity, channel half-height and kinematic viscosity, respectively. They observed that even in the limit of Newtonian flows, there are the moments of “low-drag” or “hibernating” turbulence, which display many similar features to MDR (a phenomenon generally associated with the polymer additives). They coined the nomenclature of a “hibernating” state when the flow was drag-reducing and resembles MDR, and “active” state for the rest of the flow. The major flow characteristics observed during hibernation were only weak streamwise vorticity and three-dimensionality, and lower than average wall shear stress. The frequency of these events increases with increasing viscoelasticity, although the events remain unchanged, i.e., they display similar flow properties as MDR. The connection between the polymeric drag reduction in turbulent flows and transition to turbulence in Newtonian flows has also been discussed earlier by Dubief et al. [11].

Xi and Graham [12] further investigated this phenomenon to provide detailed characteristics of active and hibernating turbulence in Newtonian and viscoelastic flows. They defined hibernation when the area-averaged wall shear stress was below 90% of the mean for a dimensionless time duration of $\Delta t^{*}=\Delta tu_{\tau }/h≳3.5$, where Δt represents the dimensional time duration. Park and Graham [13] carried out DNS for MFU in a channel flow geometry, close to transition. They obtained five families of ECS solutions, which they denoted as the “P1, P2, P3, P4 and P5” solutions. Out of these five families of solutions, “P4” solution shows the most interesting behaviour. For the upper branch solutions, the velocity profile approaches the classic von Kármán log-law, while for the lower branch solutions the velocity profile approaches the Virk’s MDR asymptote. They suggested that most of the time the turbulent trajectories remain at the upper-branch state (or the “active” state) with few excursions to the lower-branch state (or the hibernating state). This result provided a further verification that there are intervals of low-drag in Newtonian flows when the mean velocity profile is close to Virk’s MDR profile as previously observed by Xi and Graham [10,12]. The existence of such solutions for Newtonian flows has a potential application in drag reduction, which makes it a practically significant field of research.

One major characteristic of wall-bounded turbulent flows is the so-called bursting process, which is an abrupt breaking of a low-speed streak as it moves away from the wall [14]. Itano and Toh [15] investigated the bursting process for channel flow at $Re_{\tau }=130$ by computing TW solutions in a MFU using a shooting method. They observed that the bursting process is linked to the instability of the TW solution. Park et al. [16] studied the connection between the bursting process and the ECS solutions in minimal channel flow for $75\leq Re_{\tau }\leq 115$. They focussed on the P4 family of ECS solutions, as identified earlier by Park and Graham [13]. To detect a hibernating event they used the criteria that the area-averaged wall shear stress should go below 90% of the mean wall shear stress and stays there for a duration of $\Delta tU_{cl,lam}/h>65$, where $U_{cl,lam}$ is the laminar centerline velocity. This time-duration corresponds to $\Delta t^{*}>3$ for $Re_{\tau }=85$. They defined bursting events based on an increase in the volume-averaged energy dissipation rate by 50% of its standard deviation for a duration of $\Delta tU_{cl,lam}/h>15$. They observed that many of the low-drag or hibernating events are followed by strong turbulent bursts. Based on this observation, they divided the turbulent bursts into two categories: weak and strong bursts, and suggested that the strong bursts are the ones which are always preceded by a hibernating event. They also investigated the possible link between the turbulent bursts and the instability of the P4-lower branch solution. Very similar trajectories were observed for the strong bursts and the lower branch of the P4 solution, which provides further evidence that the turbulent bursts are directly related to the instability of the ECS.

Initially, the investigation of these low-drag events was conducted in minimal channels, and therefore the need was to study this phenomenon for fully turbulent flow in extended domains. The relation between the minimal channels and flow in large domains was studied by Jiménez et al. [17] and Flores and Jiménez [18]. They suggested that the flow dynamics in minimal channels have many features that are representative of fully turbulent flows. It has also been seen that some of these solutions are highly localised and display the nontrivial flow only for a small region of an extended domain, whereas the rest of the flow remains laminar [19,20,21]. Kushwaha et al. [22] carried out an investigation into these low-drag events in an extended domain for channel flow at three Reynolds numbers, $Re_{\tau }$ = 70, 85 and 100. The computational domain, in wall (or inner) units, was $L_{x}^{+}\approx 3000$ and $L_{z}^{+}\approx 800$ long in the streamwise and spanwise directions, respectively. They carried out a temporal and spatial analysis for extended domains and compared the results between the two. Regions or events of both low- and high-drag events were investigated in large domains, unlike previous MFU studies where the focus was primarily on low-drag events. To study the temporal intermittency, they employed the following criteria to detect low-drag (hibernating) or high-drag (hyperactive) events: the instantaneous wall shear stress ($\tau_{w}$) should remain below 90% or above 110% of time-averaged value for a time duration of $\Delta t^{*}=\Delta tu_{\tau }/h=3$ for low or high drag events, respectively. For studying the velocity characteristics during these low- and high-drag intervals in the flow, a conditional sampling technique was employed. They observed that, although the temporal and spatial analyses are independent of each other, the characteristics of low- and high-drag events obtained using these two methods were very similar. They found that for $Re_{\tau }$ between 70 and 100, the regions of low-drag in an extended domain show similar conditional mean velocity profiles as obtained from temporal interval of low-drag in minimal channels for $y^{+}=yu_{\tau }/\nu <30$, where *y* is the wall-normal distance. This showed that the spatiotemporal intermittency observed in extended channel flow is related to the temporal intermittency in a minimal channel.

Whalley et al. [23,24] carried out an experimental investigation of the low- and high-drag events in a plane channel flow at three Reynolds numbers, $Re_{\tau }$ = 70, 85 and 100. Instantaneous velocity, wall shear stress and flow structure measurements were conducted using laser Doppler velocimetry (LDV), hot-film anemometry (HFA) and stereoscopic particle image velocimetry (SPIV), respectively. They employed the same criteria as Kushwaha et al. [22] to detect the low-drag events, but for the high-drag events, the criteria were slightly relaxed in order to obtain more events, as the high-drag events were found to occur at a lower frequency than the low-drag events. Instantaneous velocity and wall shear stress measurements were made at the same streamwise/spanwise location, enabling conditional sampling of the velocity data to be carried out. The conditionally averaged streamwise velocity and wall shear stress were found to be highly correlated until $y^{+}\approx 40$ and a resemblance was observed between the conditionally sampled mean velocity profiles for $y^{+}≲40$ and the lower branch of the P4 ECS solution as observed earlier in minimal channels [13]. They also observed that the fraction of time spent in hibernation (low-drag) decreases with increasing Reynolds number for $70<Re_{\tau }<100$.

Recently, Pereira et al. [25] carried out DNS in channel flow of domain size, $L_{x}\times L_{y}\times L_{z}=8\;\pi h\times 2\;h\times 1.5\;\pi h$ at $Re_{\tau }$ between 69.26 and 180 for Newtonian flow, and at $Re_{\tau 0}$ = 180 for drag-reducing flow (65% drag reduction). The flow was identified as hibernating if the spatially-averaged wall shear stress was lower than 95% of its time-averaged value and no time criteria were used (unlike previous studies where a minimum time duration was also used to detect a hibernating event, for example, in [16,22,24]). They demonstrated that the transition to turbulence in Newtonian flows shares various common features to the polymer induced drag reduction in turbulent flows.

Until now, these low- and high-drag events are investigated for $70\leq Re_{\tau }\leq 100$, and therefore a natural question arises as to what are the characteristics of these events in the so-called fully-turbulent flow regime (often associated with a threshold value of $Re_{\tau }\geq 180$ [26]). The Reynolds shear stress characteristics during these events has been studied using the DNS in MFUs [12,13], yet there is no relevant experimental data or numerical data in extended domains available. In this paper, the low- and high-drag intermittencies are investigated using experimental and numerical techniques to answer these fundamental questions. The experiments are conducted in a channel flow facility using wall shear stress and velocity measurements. Recently, Agrawal et al. [27] observed that the flow in the present channel consists only of turbulent events beyond $Re_{\tau }\approx 67$ and that significant Reynolds number dependence of the skewness and flatness of wall shear stress fluctuations starts to disappear by $Re_{\tau }≃73\text{–}79$. Based on these results, in this work, the intermittences associated with the turbulent flow are investigated for $Re_{\tau }\geq 70$. An experimental study is made for Reynolds number up to $Re_{\tau }$ = 250, to probe the characteristics of these events for fully-turbulent channel flow. To study the Reynolds shear stress for $Re_{\tau }$ = 70 and 85, experimental as well numerical techniques are employed.

## 2. Experimental Set-Up

**Figure 1:**
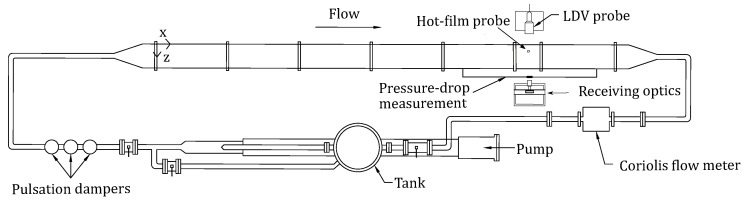
Schematic of channel-flow flow facility (not to scale).

In this study, a channel flow facility at the University of Liverpool has been utilised to carry out the experimental investigation. The same facility has been used earlier by Whalley et al. [23,24] and Agrawal et al. [27,28,29], and is shown here in Figure 1. The channel-flow facility is a rectangular duct consisting of 6 stainless steel modules and a test section. The test section is connected downstream of five stainless steel modules. Each module is of length 1.2 m and the test section has a length of 0.25 m. The width (*w*) and half-height (*h*) of the duct are 0.298 m and 0.0125 m, respectively, giving an aspect ratio (w/2h) of 11.92. The modules are constructed in such a manner as to ensure a hydraulically smooth transition between the modules.

The working fluid is stored in a stainless steel header tank of capacity about 500 L. A Mono type E101 progressive cavity pump is used to circulate the fluid via the tank in a closed loop. The flow loop also consists of an additional mixing loop which provides an opportunity for having lower flow rates. Three pulsation dampers are situated just after the pump, which helps in damping any pulsations in the flow before entering the channel. A Promass Coriolis flow meter is installed in the return loop to measure the mass flow rate ($\dot{m}$) of the fluid. This enables the bulk velocity ($U_{b}$) to be determined by the relation $U_{b}=\dot{m}/\left(\rho A\right)$, where *A* is the cross-sectional area of the channel and ρ is the density of the working fluid. A platinum resistance thermometer (PRT) is present in the last module of the channel which is used to measure the temperature of the working fluid. The PRT is powered by an Agilent 34,970 A switch unit, which provides temperature readings with a resolution of 0.01 ${}^{\circ }$C. Throughout this study, only Newtonian fluids are used as working fluids. These are water–glycerol mixtures of different concentrations where glycerol is used to increase the viscosity to get to lower Reynolds number. For example, while studying the flow for $Re_{\tau }\geq 180$, water is used as the working fluid and while studying low Reynolds number flow ($Re_{\tau }=70$), a 65%:35% by weight glycerol–water mixture is used as the working fluid. The density of the working fluid is measured using an Anton Paar DMA 35 N density meter. The shear viscosity of the working fluid is measured using an Anton Paar MCR 302 rheometer. A cone and plate geometry is employed to measure shear viscosity for shear rate $\left(\dot{\gamma },s^{-1}\right)$ ranging from $10^{-2}$ to $10^{2}$.

Pressure-drop measurements are conducted using a Druck LPX-9381 low-differential pressure transducer, which has a working range of 5 kPa with an accuracy of ±5 Pa. A Baratron differential pressure transducer made by MKS is used to regularly calibrate the Druck pressure transducer. Instantaneous wall shear stress and velocity measurements are carried out using a hot-film anemometry (HFA) system and a laser Doppler velocimetry (LDV) system, respectively, in the test section. The side- and top-walls of the test section are made of borosilicate glass to provide optical access for the LDV measurements. A Dantec FiberFlow laser system is employed for velocity measurements which uses a 300 mW argon-ion continuous wave laser. Up to two component velocity measurements have been carried out thus requiring two pairs of laser beams of different wavelengths: blue (488 nm) and green (515.5 nm). A Bragg cell is utilised to resolve the directional ambiguity of the velocity of seeding particles by giving a frequency shift of 40 MHz to one of the laser beams. The laser beams are emitted using a transmitting optics (or laser head) which provides a beam separation of 51.5 mm and a focal length of 160 mm in air. The crossing of two beams of the same colour creates a measurement volume of 24 μm diameter and 150 μm length in air. The transmitting optics is placed on a traverse which allows movement of the measurement volume in all three directions. For the seeding particles, generally, natural particles present in the working fluid (for example, supply water) are found to be sufficient to obtain a good data rate. In cases where the natural seeding particles are found to be low, for example, when the working fluid has a high concentration of glycerol, Timiron Supersilk MP-1005, having an average size of 5 μm, are added to the working fluid. In this study, both single component and two-component velocity measurements have been carried out. In the case of two-component velocity measurements, the data are acquired in co-incident mode. This mode samples both velocity components of the same seeding particle simultaneously in the measurement volume. The LDV is operated in a forward-scatter mode and the typical data rate is around 100–500 Hz. The light scattered from the seeding particle enters the photodetector (receiving optics) which splits the laser beams based on the wavelengths. The laser beams then pass to the photomultiplier tubes (PMTs) which sends the Doppler frequencies to the flow processor, burst spectrum analyzer (BSA)-F50, made by Dantec Dynamics. The signals are converted to the corresponding velocity signals using the inbuilt signal processors in the flow processor.

Calculation of RSS requires simultaneous measurements of streamwise and wall-normal velocities, but the wall-normal velocity measurements cannot be made close to the bottom wall because of the cut-off of the laser beams [30], and therefore some modifications to the transmitting optics of the LDV set-up are made. The first modification is to rotate the laser head by 45${}^{\circ }$ about the spanwise axis to get closer to the bottom wall, similarly to as previously done by Melling and Whitelaw [31], Walker and Tiederman [32] and Günther et al. [33]. Streamwise (*U*) and wall-normal (*V*) velocity components are recovered based on the coordinate transformation equation, as shown below.

(1) $$\left[\begin{array}{c} U\\ V \end{array}\right]=\left[\begin{array}{cc} \cos45^{\circ } & \sin45^{\circ }\\ -\sin45^{\circ } & \cos45^{\circ } \end{array}\right]\left[\begin{array}{c} U_{1}\\ U_{2} \end{array}\right].$$

Here, $U_{1}$ and $U_{2}$ are the velocity components measured by blue and green beams, respectively. This modification makes the minimum vertical height where the measurement of the wall-normal velocity component can be made reduced by a factor of $1/\sqrt{2}$. Next, an external LD1613-N-BK7 biconcave lens, made by Thorlabs, is placed in front of the laser head to increase the focal length of the laser beams. This lens has a diameter of 25.4 mm and a focal length of 100 mm. Increasing the focal length enables the measurement volume to go further into the test section from the side-wall. Therefore, if the aim is to measure at the same spanwise location in the test section, the laser head needs to be moved further back from the side-wall. This modification enables the laser beams to be closer to each other when they enter through the side-wall. The measurement volume can get closer to the bottom wall as the laser beams get closer to each other. Thus, the two-component velocity measurements can be carried out closer to the bottom-wall after the addition of a biconcave lens. The lens is connected on a lens mount which is attached to an optical post. The optical post is then attached to the traverse of the transmitting optics. Therefore, the entire lens system can be traversed with the transmitting optics. It is important that both pairs of laser beams are aligned properly to the external lens. This alignment is checked based on the high data rate of the LDV signal in co-incident mode and validating the time-averaged RSS profile against available DNS data at the same Reynolds number. By making these two modifications, the two-component velocity measurements can be conducted for y/h≥0.3 at a spanwise location of z/h=5 in the channel-flow facility.

In this study, constant temperature anemometry (CTA) is employed for measuring the instantaneous wall shear stress by utilising the commercially available 55R48 glue-on hot-films probes (made by Dantec Dynamics). The hot-film sensor has a physical spanwise length (Δz) of 0.9 mm. In inner units, this corresponds to $\Delta z^{+}=18$ for $Re_{\tau }=250$. In this study, the effect of measurement resolution issues due to sensor sizes are thought to be negligible as Ligrani and Bradshaw [34] considered a sensor length of about $\Delta z^{+}≲20-25$ to be acceptable to make well-resolved turbulence measurements. In order to attach the sensor to the channel wall, removable Delrin plugs are designed and fabricated inhouse. The hot-film probes are glued on these plugs and these plugs are then inserted into the bottom wall of the test section. We ensure that the hot-films are flush with the bottom wall of the test section. A detailed description of the mounting process for the hot-film probes in the present channel has been provided in Agrawal [35]. The probe is powered by a Dantec StreamLine Pro velocimetry system. The bridge ratio and the overheat ratio of the anemometer are set at 10 and 1.1, respectively. The typical frequency response of the anemometer, against the square-wave generator is found to be around 10–30 kHz, which is generally considered sufficient for turbulence measurements [36]. The output voltage signal from the anemometer is then digitized using a 14-Bit USB6009 Multifunction A/D converter, made by National Instruments. After A/D converter, the signal is acquired using the CTA application software, StreamWare Pro, installed on the computer. In the case of simultaneous measurements of velocity and wall shear stress, the digitised voltage is sampled by the BSA flow processor which helps in the acquisition of time-synchronised velocity and wall shear stress data. The voltage output signals from the anemometer is converted to instantaneous wall shear stress signals using calibration against the mean pressure-drop obtained from the pressure transducer. The same procedure for the hot-film calibration as discussed in Agrawal et al. [27,28] has been conducted here.

In CTA, all the changes in the fluctuations in voltage output from the anemometer should be representative of fluctuations in the flow. Therefore, any change in voltage output due to thermal and non-thermal drifts need to be minimised. To minimise the thermal drift, an open-loop copper cooling coil is added to the overhead tank and the main supply water is used to control the temperature of the working fluid. Using this set-up, the temperature of the working fluid could be controlled to the precision of ±0.01 ${}^{\circ }$C for the entire experimental run of the day (typically about 6–8 h). Non-thermal drifts are also observed which are generally caused due to the contamination of the hot-films [37]. A novel nonlinear regression technique, as discussed in Agrawal et al. [28], has been employed to recover the wall shear stress signals from the drifted voltage signal.

Experiments are conducted for five Reynolds numbers: $Re_{\tau }$ = 70, 85, 120, 180 and 250 and for each Reynolds number, wall shear stress and velocity data are acquired simultaneously in the measurement test section using HFA and LDV, respectively, at a location of z/h=5 and x/h=496. As discussed in Agrawal et al. [27], the spanwise location of z/h=5 is observed to be devoid of side-wall effects. Velocity acquisition is realised at various wall-normal locations, where each wall-normal location is sampled for 2 h at a typical data rate of around 300–400 Hz. Table 1 shows the Reynolds numbers, corresponding wall-normal locations studied and the parameters measured in this work. For $Re_{\tau }$ = 70 and 85, both streamwise and wall-normal velocity components are measured simultaneously with the wall shear stress. These particular measurements have been conducted to study the RSS behaviour during the low- and high-drag events. For other Reynolds numbers, due to experimental limitations, only streamwise velocity measurements have been executed along with the wall shear stress because the near peak region of the RSS could not be measured for higher Reynolds numbers as this moves physically closer to the wall at higher Reynolds numbers where the LDV beams lose optical access.

The procedure described by Kline and McClintock [38] has been employed here to conduct an uncertainty analysis of the measured and calculated variables. The employed channel-flow facility is carefully machined to provide negligible relative uncertainties (~0.15%) in the channel dimensions (*w* and *h*) and the length between the pressure tappings, *l*. The pressure transducer has an accuracy of ±5 Pa, and therefore the relative uncertainty in the mean wall shear stress is $\Delta \tau_{w}/\bar{\tau_{w}}$ = 1–3%. The density meter has a quoted accuracy of ±1 kg/m^3^. This gives a relative uncertainty in the density of the working fluid of Δρ/ρ = 0.09%. The relative uncertainty in the viscosity (μ) measurement of the working fluid using the rheometer is Δμ/μ = 2%. The relative uncertainty in the friction velocity ($u_{\tau }=\sqrt{\tau_{w}/\rho }$) is $\Delta u_{\tau }/u_{\tau }$ = 0.5–1.5%. This gives an uncertainty in the friction Reynolds number ($Re_{\tau }=u_{\tau }h/\nu$) measurement of $\Delta Re_{\tau }/Re_{\tau }$ = 2–2.5%. The major sources of error in LDV data are due to velocity gradient broadening, velocity bias effect or fringe distortion [39]. These combined effects, in general, give the relative uncertainties in the mean velocity of 2–3% and the turbulent intensities of 4–6%. In inner units, the relative uncertainties in the mean velocities and turbulent intensities are $\Delta U^{+}/U^{+}$ = 2–3.5% and $\Delta {uv}^{+}/{uv}^{+}$ = 4–7%. Here, *u* and *v* represent streamwise velocity fluctuation and wall-normal velocity fluctuation, respectively. The LDV transmitting optics traverse has a precision of 0.001 mm, providing a relative uncertainty in the wall-normal position (*y*) measurement, close to the wall (*y* = 0.5 mm), to be Δy/y = 0.2%. In inner units, at this wall-normal location, $y^{+}$ has an uncertainty of $\Delta y^{+}/y^{+}$ = 2–2.5%.

In this study, two different ways of averaging the measured variables are carried out: time-averaging and conditional-averaging. To differentiate between these two averages the following nomenclature are used: an overbar indicates a time-averaged quantity (e.g., $\bar{U}$), and an overbar with an *L* or *H* superscripts indicates the conditionally-averaged quantity for low- and high-drag events (e.g., ${\bar{U}}^{L},{\bar{U}}^{H}$), respectively. Similarly, friction velocities are calculated using two different wall shear stress: time-averaged wall shear stress ($\bar{u_{\tau }}$) and conditionally-averaged wall shear stress (${\bar{u_{\tau }}}^{L},{\bar{u_{\tau }}}^{H}$). Based on these definitions of the friction velocities, the wall-normal locations are also normalised in three different ways: $y^{+}=y\bar{u_{\tau }}/\nu$, $y^{+L}=y{\bar{u_{\tau }}}^{L}/\nu$ and $y^{+H}=y{\bar{u_{\tau }}}^{H}/\nu$.

## 3. Numerical Procedure

We consider an incompressible Newtonian fluid in the plane Poiseuille (channel) geometry, driven by a constant volumetric flux *Q*. The *x*, *y* and *z* coordinates are aligned with the streamwise, wall-normal and spanwise directions, respectively. Periodic boundary conditions are imposed in the *x* and *z* directions with fundamental periods $L_{x}$ and $L_{z}$, and a no-slip boundary condition is imposed at the walls y=±h, where $h=L_{y}/2$ is the half-channel height. The laminar centreline velocity for a given volumetric flux is given as $U_{cl,lam}=\left(3/4\right)Q/h$. Using the half-height *h* of the channel and the laminar centreline velocity $U_{cl,lam}$ as the characteristic length and velocity scales, respectively, the non-dimensionalised Navier–Stokes equations are given as

(2) ∇·u=0,

(3) $$\frac{\partial u}{\partial t}+u\cdot \nabla u=-\nabla p+1/\left(Re_{c}\right)\nabla^{2}u.$$

Here, we define the Reynolds number for the given laminar centreline velocity as $Re_{c}=U_{cl,lam}h/\nu$, where ν is the kinematic viscosity of the fluid. Characteristic inner scales are the friction velocity $u_{\tau }=\sqrt{\left(\bar{\tau_{w}}/\rho \right)}$ and the near-wall length scale, or wall unit, $\delta_{\nu }=\nu /u_{\tau }$, where ρ is the fluid density and $\bar{\tau_{w}}$ is the time- and area-averaged wall shear stress. Quantities non-dimensionalised by the inner scales are denoted with a superscript ‘+’. The friction Reynolds number is then defined as $Re_{\tau }=u_{\tau }h/\nu =h/\delta_{\nu }$. For the current simulations, friction Reynolds numbers of $Re_{\tau }$ = 70 and 85 are considered. Simulations are performed using the open source code ChannelFlow written and maintained by Gibson [40]. We focus on a domain of $L_{x}\times L_{y}\times L_{z}=13.64\;\pi h\times 2\;h\times 3.64\;\pi h$. These dimensions correspond to $L_{x}^{+}\times L_{z}^{+}\approx 3000\times 800$ for $Re_{\tau }=70$, and $L_{x}^{+}\times L_{z}^{+}\approx 3640\times 970$ for $Re_{\tau }=85$. A numerical grid system is generated on $N_{x}\times N_{y}\times N_{z}$ (in *x*, *y*, and *z*) meshes, where a Fourier–Chebyshev–Fourier spectral spatial discretisation is applied to all variables. A resolution of ($N_{x},N_{y},N_{z}$) = (196, 73, 164) is used for both Reynolds numbers. The numerical grid spacing in the streamwise and spanwise direction are $\Delta x_{min}^{+}\approx 15.3$ (18.6) and $\Delta z_{min}^{+}\approx 4.9\left(5.9\right)$ for $Re_{\tau }$ = 70 and ($Re_{\tau }$ = 85) cases. The nonuniform Chebyshev spacing used in the wall-normal direction results in $\Delta y_{min}^{+}\approx 0.07$ (0.08) at the wall and $\Delta y_{max}^{+}\approx$ 3.0 (3.7) at the channel centre for $Re_{\tau }$ = 70 and ($Re_{\tau }$ = 85) cases. For the computation time, 50 × $10^{3}$ strain times ($>25Re_{c}$) is chosen to attain meaningful statistics.

The present experiment provides temporal information for the flow, and therefore for a comparison of the DNS and experimental data, temporal information from the DNS data is extracted. To obtain reliable statistics, nine wall locations are chosen at the wall on the top and on the bottom walls of the computational domain. These locations are selected in such a way that each spatial location is not correlated with the others [22]. The streamwise/spanwise spatial locations correspond to the combinations of three $x^{+}$ locations and three $z^{+}$ locations: $x^{+}$≈ 505, 1500 and 2495; $z^{+}$≈ 151, 400 and 649 for $Re_{\tau }$ = 70, and $x^{+}$≈ 613, 1820 and 3027; $z^{+}$≈ 183, 485 and 787 for $Re_{\tau }$ = 85. The instantaneous wall shear stress is obtained by using the streamwise velocity gradient information at $y^{+}\approx 1$, although no difference in its value was observed between $y^{+}\approx 1$ and lower $y^{+}$ locations.

## 4. Identifying Low- and High-Drag Events

**Figure 2:**
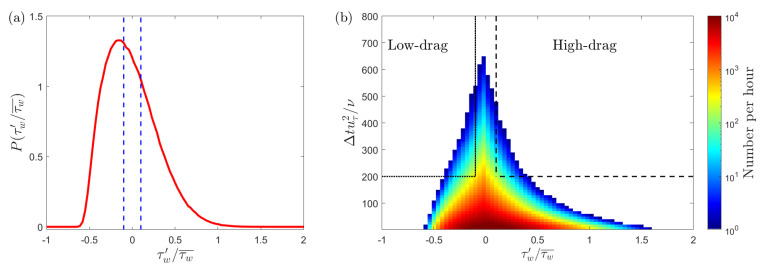
(**a**) PDF of wall shear stress fluctuations for $Re_{\tau }=$ 180 obtained using experiments. Blue dashed lines represent the threshold criteria for the low- and high-drag events, i.e., $\tau_{w}/\bar{\tau_{w}}<$ 0.9 and $\tau_{w}/\bar{\tau_{w}}>$ 1.1, respectively. (**b**) Distribution of negative and positive wall shear stress fluctuations per hour for $Re_{\tau }=$ 180 obtained using experiments. The black dotted lines cover the region of low-drag events based on the criteria: $\tau_{w}/\bar{\tau_{w}}<$ 0.9 and $\Delta t_{cr}^{+}=200$ and the black dashed lines cover the region of high-drag events based on the criteria: $\tau_{w}/\bar{\tau_{w}}>$ 1.1 and $\Delta t_{cr}^{+}=200$.

Figure 2a shows the PDF (probability density function) of wall shear stress fluctuations ($\tau_{w}^{\prime }$) obtained at $Re_{\tau }=180$ using experiments. The PDF of wall shear stress has a longer positive tail which means that the PDF is positively skewed. This shows that some of the positive fluctuations have much larger magnitude than the negative fluctuations. In the present study, the wall shear stress is representative of the skin-friction drag. Previously, Gomit et al. [41] used the PDF of wall shear stress to divide low- and high-wall shear stress events in a turbulent boundary layer. They divided the PDF into four quartiles, where each quartile contains one-fourth of the realisations. In this study, to define the low- and high-drag “events”, two significant parameters are considered: the magnitude of the wall shear stress fluctuations and the duration of time the fluctuations stay below or above the time-averaged value.

The PDF of wall shear stress fluctuations, as shown in Figure 2a, provides statistical information about the magnitude of the fluctuations but information regarding the time-duration of the fluctuations cannot be inferred. Therefore, it is necessary to find a way to visualise all the positive and negative fluctuations as a function of the magnitude and time-duration. This is carried out by calculating the distribution of all the fluctuations ($\tau_{w}^{\prime }$) about the time-averaged value ($\bar{\tau_{w}}$) with their corresponding time durations (Δt). Figure 2b shows this distribution for $Re_{\tau }$ = 180. Here, inner scaling ($u_{\tau }^{2}/\nu$) is used to scale the time-duration of the negative and positive wall shear stress fluctuations. The strength of the wall shear stress fluctuations is given by $\tau_{w}^{\prime }/\bar{\tau_{w}}$. The number of these fluctuations is higher for the lower strengths and lower time-durations.

**Figure 3:**
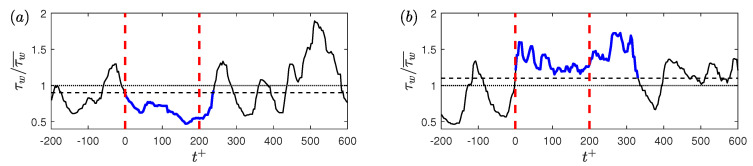
Time history of normalised wall shear stress at $Re_{\tau }=180$ during (**a**) a low-drag and (**b**) a high-drag event obtained using experiments. Blue solid lines highlight the low-drag and the high-drag events in panels (**a**,**b**), respectively. Black dotted lines show mean value of normalised wall shear stress $\tau_{w}/\bar{\tau_{w}}=1$. Black dashed lines show $\tau_{w}/\bar{\tau_{w}}=0.9$ and $\tau_{w}/\bar{\tau_{w}}=1.1$ in panels (**a**,**b**), respectively. Red dashed line indicates the time-duration criteria of $\Delta t_{cr}^{+}$ = 200. In panels (**a**,**b**), $t^{+}$ is shifted such that $t^{+}$ = 0 indicates the beginning of a conditional event.

In this study, to detect a low-drag or a high-drag event, a magnitude threshold criterion and a time duration criterion are employed on the wall shear stress signals. For the threshold criteria, values less than 0.9$\tau_{w}$ for the low-drag events and greater than 1.1$\tau_{w}$ for the high-drag events have been typically employed previously by Kushwaha et al. [22]. Whalley et al. [24] used the same threshold criteria for the low-drag events, but for the high-drag events they employed a less stringent criteria of greater than 1.05$\tau_{w}$, in order to obtain more data points to carry out the statistical analysis. In the present study, the same values for the threshold criteria as used by Kushwaha et al. [22] are employed to detect the conditional events; however, the effect of varying the threshold criteria will also be discussed. For the time-duration criteria, Kushwaha et al. [22] and Whalley et al. [23,24] employed a mixed scaling ($\Delta t^{*}=\Delta tu_{\tau }/h$) to detect conditional events in channel flows. They typically used $\Delta t^{*}=3$ as the time-duration criterion while discussing the sensitivity of the value of the time-duration criterion on the conditional quantities. Unlike these previous studies, in the present investigation, an inner scaling is used for the time-duration criterion for the conditional events: $\Delta t^{+}=200$ is used as the minimum time-duration to detect conditional events. The reasons for, and implications of, choosing this scaling will be discussed in detail in the next section. The effect of varying the length of the time-duration criterion on the conditional quantities will be discussed in Section 6. To further understand the definition of these conditional events, examples of instantaneous wall shear stress signals meeting the above-mentioned criteria for the low-drag and the high-drag events are shown in Figure 3. This figure shows the instantaneous normalised wall shear stress during the low-drag (Figure 3a) and the high-drag (Figure 3b) events. In Figure 3, the acquisition time of the wall shear stress is shifted such that $t^{+}=0$ indicates the beginning of a low- or a high-drag event. Each event is shown to act longer than the minimum time duration (for “low-drag” ~230 units and for “high-drag” ~320 units).

## 5. Time Spent in Low- and High-Drag Events

Here we study the effect of three different scalings, i.e., inner scaling, mixed scaling and outer scaling for the time-duration criteria to detect a conditional event. Outer scaling is simply $\Delta tU_{b}/h$. Inner scaling ($\Delta t^{+}=\Delta t\frac{u_{\tau }^{2}}{\nu }$) and the mixed scaling ($\Delta t^{*}=\Delta t\frac{u_{\tau }}{h}$) are related by the following relation.

(4) $$\Delta t^{+}=Re_{\tau }\Delta t^{*}.$$

From Equation (4), it can be observed that with increasing Reynolds numbers, the $\Delta t^{+}$ value increases for the same $\Delta t^{*}$ value. Whalley et al. [24] studied the fraction of time spent in low- and high-drag events with changing Reynolds numbers where the time-duration criterion was kept constant in mixed scaling. They observed that with increasing Reynolds number between $70\leq Re_{\tau }\leq 100$, the fraction of time spent in low-drag events decreases by approximately 500% while increasing the $Re_{\tau }$ from 70 to 100. The effect of other scalings has not been considered previously.

The fraction of time spent in the conditional events is investigated for $Re_{\tau }=$ 70, 85, 120, 180 and 250 using all three scalings. For $Re_{\tau }=70$, $\Delta tu_{\tau }/h$ = 3 corresponds to about $tu_{\tau }^{2}/\nu$ = 200 and $tU_{b}/h=42$. Based on this information, three values are chosen for each scaling to study the effect of Reynolds number on the fraction of time spent in the conditional events. For the mixed scaling, $\Delta tu_{\tau }/h$ = 1, 2 and 3, for outer scaling, $tU_{b}/h$ = 15, 30 and 45, and for the inner scaling, $tu_{\tau }^{2}/\nu$ = 100, 200 and 300 are used. For the low-drag events the threshold criterion is kept constant as $\tau_{w}/\bar{\tau_{w}}<$ 0.9 and for the high-drag events the threshold criterion is kept constant as $\tau_{w}/\bar{\tau_{w}}>$ 1.1.

**Figure 4:**
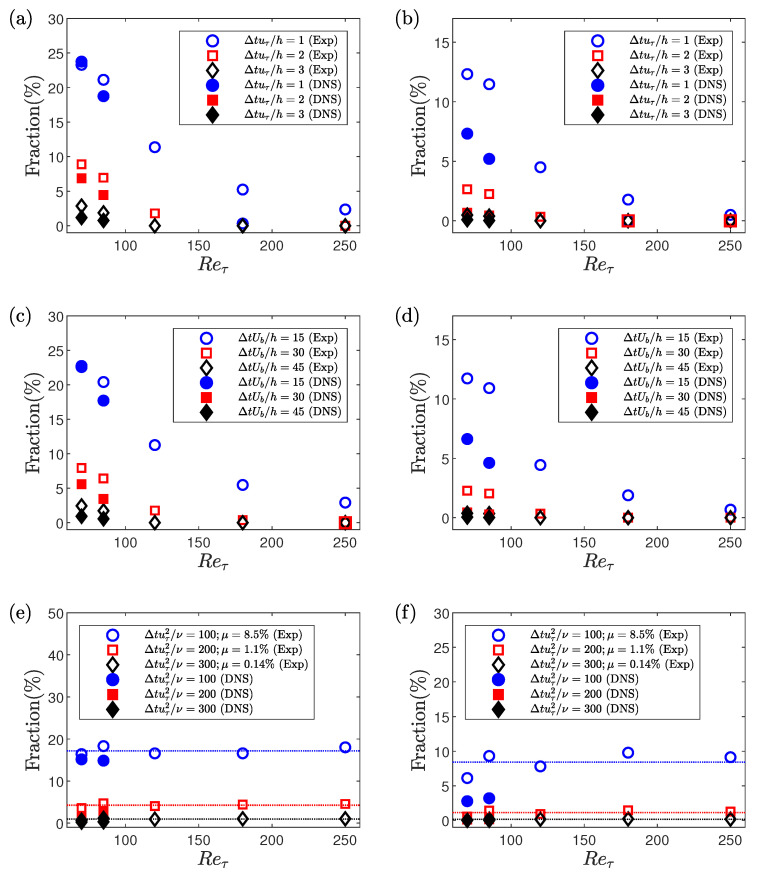
Reynolds number variation of fraction of time spent in low-drag events with using (**a**) mixed scaling, (**c**) outer scaling and (**e**) inner scaling for the time-duration criteria. Reynolds number variation of fraction of time spent in high-drag events with using (**b**) mixed scaling, (**d**) outer scaling and (**f**) inner scaling for the time-duration criteria. Open symbols represent the experimental data and filled symbols represent the DNS data. The threshold criteria to detect a low- and high-drag event are $\tau_{w}/\bar{\tau_{w}}<$ 0.9 and $\tau_{w}/\bar{\tau_{w}}>$ 1.1, respectively. Note that the *y*-axis is not the same between low- and high-drag data. Error bars obtained by dividing the sample size into two halves and calculating the respective fraction are found to be within the size of the symbols and are therefore removed to avoid cluttering of data. Dotted lines in panels (**e**,**f**) highlight the average value of fraction (%) for $70\leq Re_{\tau }\leq 250$ at different values of $\Delta tu_{\tau }^{2}/\nu$ obtained using experiments.

Figure 4 shows the fraction of time spent in low- and high-drag for different Reynolds numbers and the time-duration criteria. Results are shown for both the experimental as well as DNS data. It can be observed that the fraction of time spent in low-drag or high-drag decreases with increasing Reynolds numbers when mixed or outer scaling is used for the time duration criteria. This is similar to the result obtained using the mixed scaling for the time-duration criteria by Whalley et al. [24]. However, the fraction of time spent in the conditional events remains almost independent of the Reynolds number for $70\leq Re_{\tau }\leq 250$ for the experimental data, when the time-duration criteria is kept constant in inner units. DNS data shows a qualitatively consistent behaviour (i.e., show a similar trend for all three scalings) in the fraction of the conditional events compared to the experimental data although for a smaller range of Reynolds numbers. One possibility for the differences observed between DNS and experiments here is that these very rare low- or high-drag events involve flow structures that are much longer in the streamwise direction than usual, and that a domain size that is adequate for the vast majority of the turbulent dynamics might not be long enough to quantitatively capture the frequency of these rare events. Alternatively, subtle differences caused by the finite aspect ratio of the experimental set-up in comparison to the periodic boundary conditions used in the simulations, or the inherent uncertainties associated with the calibration of the hot-film signals maybe the cause of these differences. Based on this observation, inner scaling is chosen for the time-duration criteria in the remainder of this paper. Figure 4e,f also shows that increasing the value of the time-duration criteria (100 $\leq \Delta tu_{\tau }^{2}/\nu \leq$ 300) decreases the fraction of time spent in these conditional events. The fraction of time spent in the intervals of low-drag is found to be greater than the intervals of high-drag for the same values of the time-duration criteria for 100 $\leq \Delta tu_{\tau }^{2}/\nu \leq$ 300, and where the threshold criteria is kept the same in terms of the magnitude ($\tau_{w}/\bar{\tau_{w}}<$ 0.9 for the low-drag events and $\tau_{w}/\bar{\tau_{w}}>$ 1.1 for the high-drag events).

A similar observation was also made previously by Whalley et al. [24] while using mixed scaling for the time-duration criterion.

**Figure 5:**
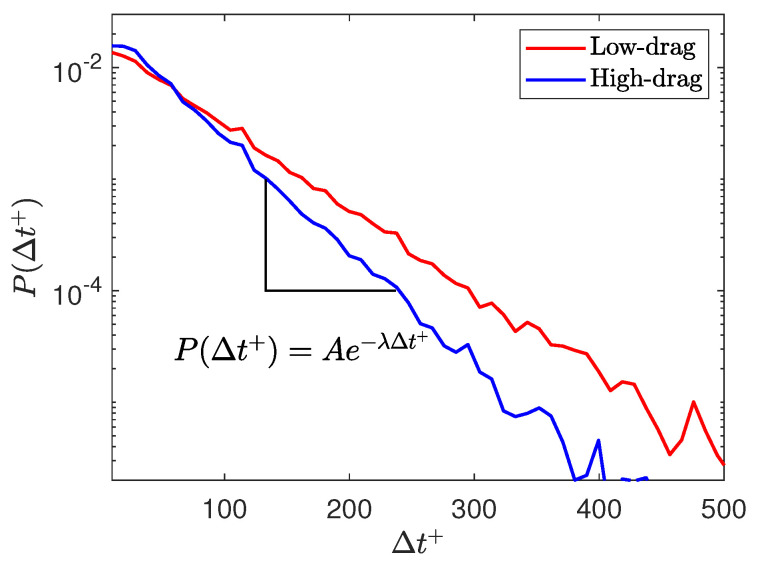
PDF of occurrence of low- and high-drag events as a function of $\Delta t^{+}$ for $Re_{\tau }=180$ where the threshold criteria for low- and high-drag events are $\tau_{w}/\bar{\tau_{w}}<0.9$ and $\tau_{w}/\bar{\tau_{w}}>1.1$, respectively. Here, x-axis ($\Delta t^{+}$) represents the lifetime or duration of a conditional event.

Figure 4 shows that the fraction of time spent in the conditional events decreases with increasing the value of the time-duration criterion. A further investigation of this phenomenon is made by studying the dependence of the occurrence of conditional events as a function of their durations. Figure 5 shows the distribution of the occurrence of low- and high-drag events as a function of $\Delta t^{+}$ for $Re_{\tau }=180$. The threshold criteria to detect a low- and high-drag events are $\tau_{w}/\bar{\tau_{w}}<0.9$ and $\tau_{w}/\bar{\tau_{w}}>1.1$, respectively. The probability of occurrence of both low- and high-drag events decreases almost exponentially (as the *y*-axis is in log scale) with increasing $\Delta t^{+}$. For $\Delta t^{+}≳400$, $P(\Delta t^{+})$ does not seem to be well resolved because of the lower occurrence of low- and high-drag events for higher $\Delta t^{+}$, thus leading to lower number of events to carry out the statistical analysis. The distribution of high-drag events is observed to be different to the distribution of low-drag events. There is a higher probability of occurrence of high-drag events for lower $\Delta t^{+}$ as compared to the low-drag events and vice versa. The crossover $\Delta t^{+}$, where the behaviour of the low- and high-drag events becomes opposite, is about 60. The decay of the probability of the low- and high-drag events is then fitted with an exponential relationship for $100\leq \Delta t^{+}\leq 300$, given by $P\left(\Delta t^{+}\right)=Ae^{-\lambda \Delta t^{+}}$. Here, λ indicates the rate of decay. The decay rate is calculated for all the Reynolds numbers. Exponential distributions like this arise in so-called Poisson processes, also called memoryless processes. The exponential decay implies that the probability of the interval ending between time $\Delta t^{+}$ and time $\Delta t^{+}+d\left(\Delta t^{+}\right)$ is independent of $\Delta t^{+}$, i.e., the probability of the low- or high-drag intervals ending are independent of how long they have lasted. Avila et al. [42] observed a similar memoryless process with regards to puff splitting during transition in a pipe flow. After an initial formation time, the distribution of puff splitting were exponential and therefore memoryless, thus showing that the probability of a puff splitting does not depend on its age. Table 2 shows the rate of decay obtained for low- and high-drag events at various Reynolds numbers. The rate of decay is found to be almost independent of the Reynolds numbers for both low- and high-drag events, and the λ values are lower for the low-drag than the high-drag for the $100\leq \Delta t^{+}\leq 300$. A slight discrepancy is observed for $Re_{\tau }=70$, which can be attributed to the presence of transitional effects at this Reynolds number, as discussed in Agrawal et al. [27]. These results are also consistent with the results shown in Figure 4e,f that the fraction of the conditional events are almost independent of the Reynolds number and the fraction of time spent in low-drag events is higher than for the high-drag events. This is the first evidence that the “low-drag” hibernating turbulent events exist significantly above the Reynolds numbers close to transition [24] and well into the regime where the flow is usually considered to be “fully-turbulent”, i.e., $Re_{\tau }\geq 180$ [26].

## 6. Wall Shear Stress Statistics during Conditional Events

**Figure 6:**
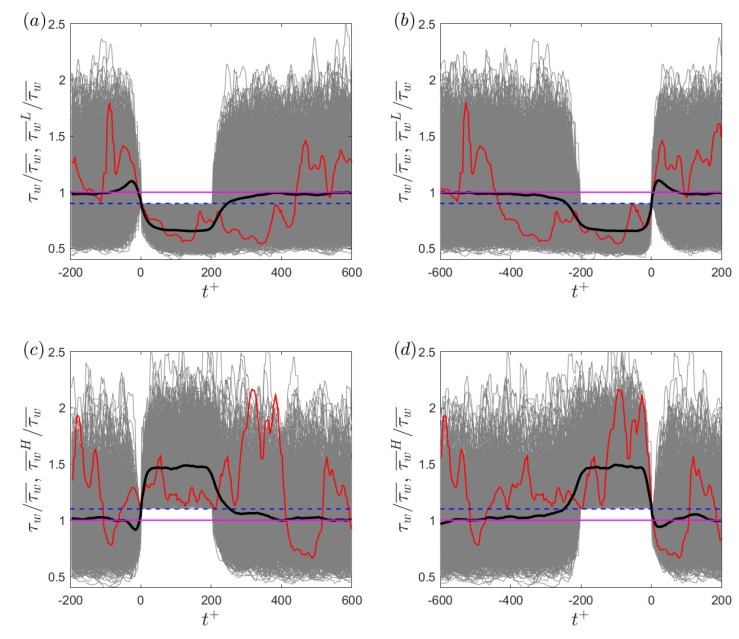
(**a**,**b**) Instantaneous normalised wall shear stress (thin grey lines) and ensemble-averaged wall shear stress (thick black line) during the low-drag events for $Re_{\tau }$ = 180 where $t^{+}=0$ indicates (**a**) start of a low-drag event and (**b**) end of a low-drag event. Red line highlights an instantaneous low-drag event with a duration of $\Delta t^{+}\approx 410$. Purple line and dashed blue line represent the time-averaged value and the threshold value of $\tau_{w}/\bar{\tau_{w}}<0.9$, respectively. (**c**,**d**) Instantaneous normalised wall shear stress (thin grey lines) and ensemble-averaged wall shear stress (thick black line) during the high-drag events for $Re_{\tau }$ = 180 where $t^{+}=0$ indicates (**a**) start of a high-drag event and (**b**) end of a high-drag event. Red line highlights an instantaneous low-drag event with a duration of $\Delta t^{+}\approx 400$. Purple line and dashed blue line represent the time-averaged value and the threshold value of $\tau_{w}/\bar{\tau_{w}}>1.1$, respectively.

To study the statistics of the conditional wall shear stress, the instantaneous wall shear stress during the low-drag or high-drag events are ensemble-averaged. Figure 6 shows the instantaneous and ensemble averaged wall shear stress fluctuations during low- and high-drag events for $Re_{\tau }=180$. The ensemble averaging is executed in two ways: by shifting all the instantaneous low- and high-drag events such that $t^{+}=0$ indicates the beginning of a conditional event (shown in Figure 6a,c), and by shifting all the instantaneous low- and high-drag events such that $t^{+}=0$ indicates the end of a conditional event (shown in Figure 6b,d). This has been done to study the time evolution of the ensemble-averaged wall shear stress with respect to the start and the end of a conditional event. It can be seen that during the low-drag events, the ensemble averaged wall shear stress drops approximately 35% below the time-averaged value. During the high-drag events, the ensemble averaged wall shear stress rises approximately 45% above the time-averaged value. This figure also highlights that although the time-duration criteria for the conditional events is $\Delta t_{cr}^{+}=200$, these events can last up to $\Delta t^{+}\geq 400$.

**Figure 7:**
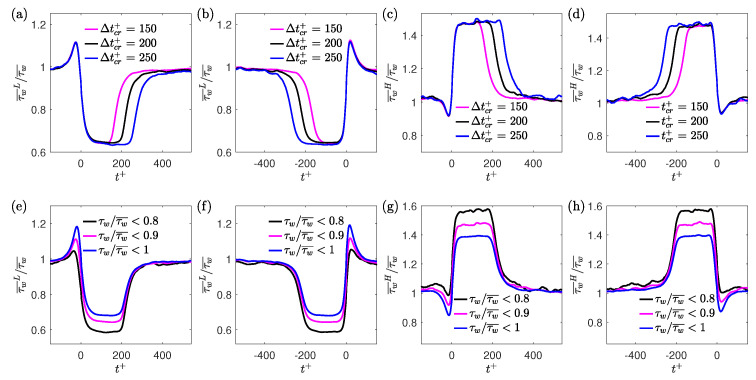
Ensemble-averaged wall shear stress for various time-duration criteria at $Re_{\tau }$ = 180 for (**a**) start and (**b**) end of low-drag events, and (**c**) start and (**d**) end of high-drag events. The threshold criteria to detect a low- and high-drag event are $\tau_{w}\;/\bar{\tau_{w}}<0.9$ and $\tau_{w}\;/\bar{\tau_{w}}>1.1$, respectively. Ensemble-averaged wall shear stress for various threshold criteria at $Re_{\tau }$ = 180 for (**e**) start and (**f**) end of low-drag events, and (**g**) start and (**h**) end of high-drag events. The time-duration criteria to detect a low-drag or a high-drag event is kept constant at $\Delta t_{cr}^{+}=200$.

The effect of the time-duration and magnitude threshold criteria on the conditional wall shear stress is investigated for $Re_{\tau }$ = 180. For the time-duration criterion, $\Delta t_{cr}^{+}$ is varied between 150 and 250 while keeping the threshold criteria constant as $\tau_{w}/\bar{\tau_{w}}<0.9$ and $\tau_{w}/\bar{\tau_{w}}>1.1$ for the low- and high-drag events, respectively. Figure 7a–d shows the ensemble-averaged wall shear stress for the low- and high-drag events at $Re_{\tau }=180$ for various time-duration criteria. The figure shows the ensemble-averaged wall shear stress for the conditional events for both methods of ensemble averaging, i.e., $t^{+}=0$ indicates either the start or end of a conditional event. The plateau of the ensemble-averaged wall shear stress during the low- and high-drag events is observed to be insensitive to the time-duration criteria when varying $\Delta t^{+}$ from 150 to 250, but the duration of these conditional events itself becomes smaller when making the criteria less stringent. A spike in the ensemble-averaged wall shear stress can be observed near the start and end of the low-drag events and similarly, a dip can be seen near the start and end of the high-drag events. Analogous results corresponding to the ensemble-averaged wall shear stress during the low-drag events were also obtained by Kushwaha et al. [22] in channel flow using DNS for $Re_{\tau }=100$. They employed mixed scaling ($\Delta t^{*}=$ 2 and 3) as the time-duration criteria to detect low-drag events. Similar results were obtained for the other Reynolds numbers studied here and are not shown for brevity.

It can be said that the time-duration criteria, either based on mixed or inner scaling (for the range studied), does not affect the strength of the low- or high-drag events. For the rest of this paper, the time-duration criteria for the both low- and high-drag events is fixed at $\Delta t_{cr}^{+}=200$ unless stated otherwise. Next, the effect of changing the threshold criteria on the conditional wall shear stress is investigated while keeping the time-duration criterion constant at $\Delta t_{cr}^{+}=200$. The threshold criteria used for low-drag events are $\tau_{w}/\bar{\tau_{w}}<0.8$, $\tau_{w}/\bar{\tau_{w}}<0.9$ and $\tau_{w}/\bar{\tau_{w}}<1$, and for the high-drag events are $\tau_{w}/\bar{\tau_{w}}>1$, $\tau_{w}/\bar{\tau_{w}}>1.1$ and $\tau_{w}/\bar{\tau_{w}}>1.2$. The most stringent limits for the strength in the threshold criteria are chosen based on the availability of a sufficient number of conditional events to obtain well-resolved ensemble-averaged wall shear stress results. As the threshold criterion is made more stringent, for the low-drag events (shown in Figure 7e,f), the lower plateau of the ensemble-averaged wall shear stress decreases. Similarly, for the high-drag events (shown in Figure 7g,h), the upper plateau of the ensemble-averaged wall shear stress increases. Similar results were observed for low-drag events only by Kushwaha et al. [22] at $Re_{\tau }$ = 100. The results are shown only for $Re_{\tau }=180$ as very similar results were obtained for the other Reynolds numbers studied.

Interestingly, as can be seen from Figure 7e–h, the spike in the ensemble-averaged wall shear stress for the low-drag events and dip in the ensemble-averaged wall shear stress for the high-drag events seems to be less significant with increasingly strict threshold criteria. Kushwaha et al. [22] mentions that they have no physical explanation for the existence of the spike or dip in the ensemble-averaged wall shear stress data. To investigate the reason for the spike or dip in the ensemble-averaged data during the conditional events, two artificially generated time series have been produced where one signal is Gaussian and the other signal has the same first four moments as the wall shear stress moments for $Re_{\tau }=180$ obtained in the present experiment. The Gaussian signal has a rms value the same as the wall shear stress for $Re_{\tau }=180$. This has been conducted to understand if the reason for the spike or the dip is unique to the wall shear stress signals or is merely a statistical artefact of the conditioning. An equal number of samples (*N* = 2 × 10^8^) are generated for both of the artificially generated signals using the inbuilt MATLAB function: “pearsrnd”.

**Figure 8:**
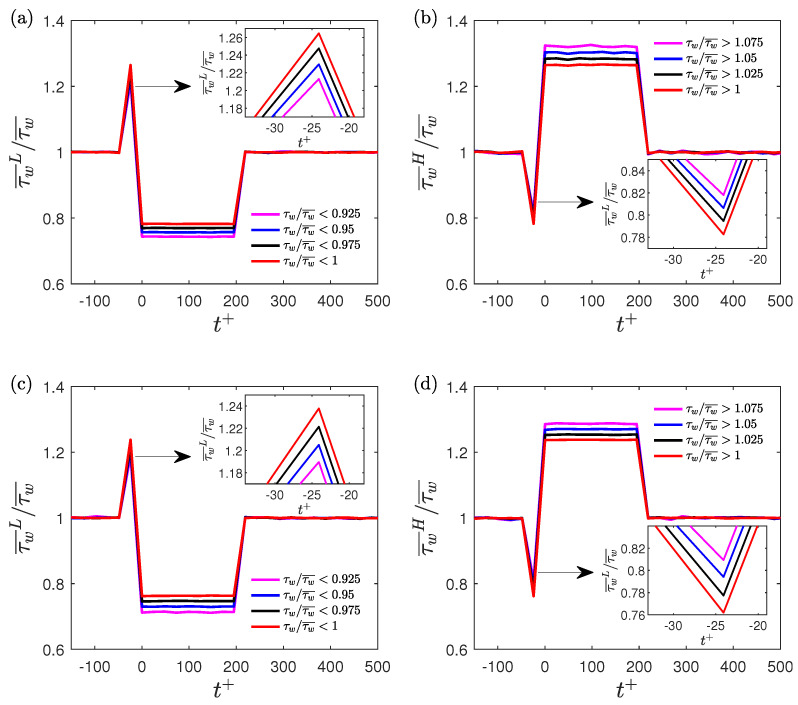
Ensemble-averaged wall shear stress during (**a**) low-drag events and (**b**) high-drag events for the artificially generated wall shear stress signal with same first four moments as one measured for $Re_{\tau }=180$. Ensemble-averaged wall shear stress during (**c**) low-drag events and (**d**) high-drag events for a Gaussian signal. The time-duration criteria to detect a low-drag or a high-drag event is kept constant at $\Delta t_{cr}^{+}=200$. Inset plots show the same data as the main plot but only near the spike or dip in the ensemble averaged data.

A comparison of the ensemble averaged data during the conditional events is made between the two artificially generated signals. The time duration is kept the same as $\Delta t_{cr}^{+}=200$ to detect the low- and high-drag events. The threshold criteria are varied to study their effect on the ensemble averaged values. For the low-drag events, the threshold criteria are $\tau_{w}/\bar{\tau_{w}}<0.925$, $\tau_{w}/\bar{\tau_{w}}<0.95$, $\tau_{w}/\bar{\tau_{w}}<0.975$ and $\tau_{w}/\bar{\tau_{w}}<1$, and for the high-drag events, the threshold criteria are $\tau_{w}/\bar{\tau_{w}}>1$, $\tau_{w}/\bar{\tau_{w}}>1.025$, $\tau_{w}/\bar{\tau_{w}}>1.05$ and $\tau_{w}/\bar{\tau_{w}}>1.075$. Figure 8 shows the ensemble averaged wall shear stress during low- and high-drag events obtained from the two artificially generated signals. There is a spike (and dip) in the ensemble-averaged wall shear stress near the start of the low-drag (and high-drag) events for both artificially generated signals. The existence of spikes or dips in the ensemble-averaged data from the artificially-generated signals, even in the limit of a Gaussian signal, suggest that these are artefacts of the conditional sampling and ensemble averaging and are not unique to the wall shear stress signals. It is also seen that the spikes (and dips) in the ensemble-averaged data from the low-drag events (and high-drag events) becomes less significant when making the threshold criteria more stringent. This further reinforces the idea that these spikes and dips in the ensemble averaged data are the consequence of the conditional sampling of any time-series signal. Thus, these spikes or dips cannot be used to identify the onset/footprint of low- or high-drag events. Park et al. [16], using MFU simulations, observed that many of the low-drag events are followed by strong turbulent bursts which were detected based on an increase in the volume-averaged energy dissipation rate. There may exist a relation between these turbulent bursts and spikes in the ensemble-averaged wall shear stress data after low-drag events which needs further investigation.

## 7. Velocity Characteristics during Conditional Events

As mentioned in Section 2, simultaneous measurements of velocity using LDV above the hot-film are made for various wall-normal locations (shown in Table 1) at every Reynolds numbers studied. However, the wall-normal velocities were measured only for $Re_{\tau }$ = 70 and 85, due to the limited access of the laser beams for LDV measurements closer to the bottom wall of the channel. Velocity information is also obtained using DNS in large computation domains (discussed in Section 3) for $Re_{\tau }$ = 70 and 85. In this section, the criteria for conditional events are kept constant at $\Delta t_{cr}^{+}=200$ and $\tau_{w}/\bar{\tau_{w}}<$ 0.9 for the low-drag events, and $\Delta t_{cr}^{+}=200$ and $\tau_{w}/\bar{\tau_{w}}>$ 1.1 for the high-drag events, unless stated otherwise. To carry out the conditional sampling of the velocity data, we ensured that there are a sufficient number of conditional events (∼100) to obtain well-converged results. For the DNS, the number of high-drag events obtained were quite few in number, between 10 and 20 for both $Re_{\tau }$ = 70 and 85. Therefore, the characteristics of only low-drag events are studied for the DNS data, whereas characteristics of both low-drag and high-drag events are studied using the experimental data.

### 7.1. Streamwise Velocity

**Figure 9:**
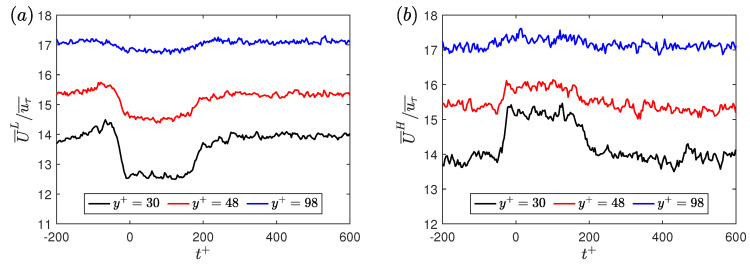
Ensemble-averaged streamwise velocity for $Re_{\tau }$ = 180 during (**a**) low-drag events and (**b**) high-drag events. Here, $t^{+}=0$ indicates the beginning of a low-drag or a high-drag event. The criteria to detect a low-drag event are $\Delta t_{cr}^{+}=200$ and $\tau_{w}/\bar{\tau_{w}}<$ 0.9, and a high-drag event are $\Delta t_{cr}^{+}=200$ and $\tau_{w}/\bar{\tau_{w}}>$ 1.1.

The conditional sampling of the velocity data and their ensemble-averaging is conducted in a similar manner as has been conducted earlier by Whalley et al. [24] and Kushwaha et al. [22]. For the low-drag events, the drop in the ensemble averaged velocities is observed to be more significant near the wall, with the effect disappearing near the centreline. For the high-drag events, an analogous behaviour to low-drag events is observed. Figure 9 shows an example of the ensemble averaged streamwise velocities for various wall-normal locations at $Re_{\tau }$ = 180 during the low- and high-drag events. Here, the ensemble-averaged streamwise velocities (${\bar{U}}^{L},{\bar{U}}^{H}$) are normalised by $\bar{u_{\tau }}$. Very similar results were observed for other Reynolds numbers and therefore are not shown. This behavior of the ensemble averaged streamwise velocities is similar to those previously obtained by Whalley et al. [24] and Kushwaha et al. [22] for $70\leq Re_{\tau }\leq 100$. Therefore, it can be said that the ensemble-averaged streamwise velocity during the low- and high-drag events, which were previously observed for $70\leq Re_{\tau }\leq 100$, shows similar characteristics even for the flow in the fully-turbulent regime.

**Figure 10:**
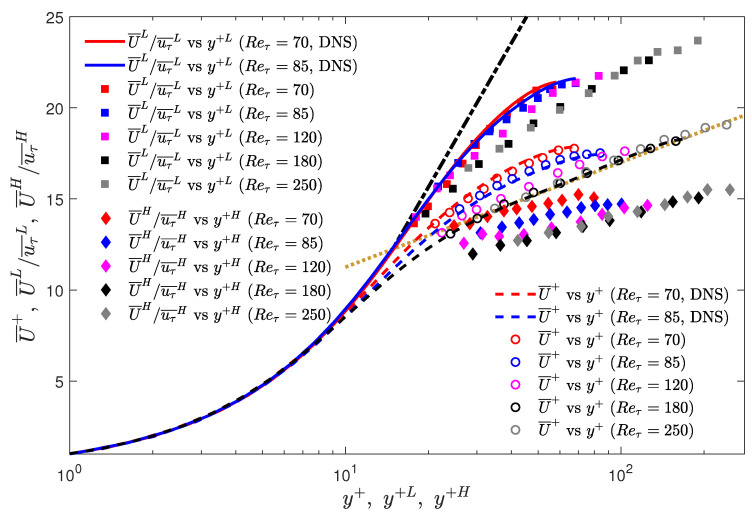
Unconditional and conditionally averaged streamwise velocity profiles for $Re_{\tau }$ = 70, 85, 120, 180 and 250 during low-drag and high-drag events. All the symbols represent the experimental data. Here, the conditionally averaged streamwise velocity data is normalised using conditionally averaged friction velocity. Yellow dotted line represents the Prandtl-von Kármán log-law: $U^{+}$ = 2.5 ln $y^{+}+5.5$ and the black dash-dotted line represents the lower end of the 95% confidence interval of the Virk’s MDR asymptote: $U^{+}$ = 11.4 ln $y^{+}-18.5$ [43]. Black dashed line represents the time-averaged velocity profile obtained using DNS at $Re_{\tau }$ = 180 by Kim et al. [26].

Figure 10 shows the unconditional and conditionally-averaged streamwise velocity profiles for $Re_{\tau }$ = 70, 85, 120, 180 and 250 obtained using experiments, and $Re_{\tau }$ = 70 and 85 obtained using DNS. Here, the normalisation of the unconditional velocity and the corresponding wall-normal locations are carried out using the time-averaged friction velocity ($\bar{u_{\tau }}$). The conditionally-averaged streamwise velocities and the corresponding wall-normal locations are normalised by the conditionally-averaged friction velocities (${\bar{u_{\tau }}}^{L}$ for low-drag and ${\bar{u_{\tau }}}^{H}$ for high-drag). Before studying the profiles during the conditional events, we first focus on the unconditional (time-averaged) profiles. Experimental and DNS results are in good agreement for $Re_{\tau }$ = 70 and 85. The unconditional profile obtained for $Re_{\tau }=180$ is also in good agreement with the DNS profile obtained by [26] for $Re_{\tau }=180$, and the velocity profiles for $Re_{\tau }$ of 180 and 250 approximately collapses on the log-law profile ($U^{+}$ = 2.5 ln $y^{+}$ + 5.5) for $y^{+}\geq$ 30.

The velocity statistics during the conditional events is investigated in such a way that only the upper (for high-drag) or lower plateau (for low-drag) of the instantaneous wall shear stress and velocity are considered for the conditional sampling. This is done to avoid any transient behaviours (start and end of conditional events) affecting the result. Therefore, only wall shear stress and velocity data between $30<t^{+}<t_{end}^{+}-30$ are used for conditional sampling, where $t_{end}^{+}$ indicates the end of a low-drag or a high-drag event. For $y^{+}≲10$, the unconditional and conditional profiles for $Re_{\tau }$ = 70 and 85 obtained using DNS almost collapse on each other. For $y^{+}≳$ 10, the conditionally averaged velocity profiles are closer to Virk’s MDR asymptote than their time-averaged values (for all the Reynolds numbers studied). Previously, Kushwaha et al. [22] and Whalley et al. [24] showed that at $70\leq Re_{\tau }\leq 100$, the low-drag velocity profiles get closer to the Virk’s MDR and the lower-branch of the nonlinear TW solutions (as obtained by Park and Graham [13]) for similar wall-normal locations, $y^{+}≲$ 35. Therefore, the present result confirms the validity of this phenomenon for Reynolds numbers in the fully-turbulent regime. There is a very good agreement between the experimental and DNS results for the velocity profiles during the low-drag events at $Re_{\tau }$ = 70 and 85. For higher wall-normal locations the conditional velocity profiles start to deviate from Virk’s MDR profile, and for $y^{+}≳100$, the conditional velocity profiles have a slightly higher slope as compared to the Prandtl-von Kármán log-law, as seen for $Re_{\tau }$ = 180 and 250. For the high-drag events, the conditional velocity profiles are lower than the unconditional profiles for all the Reynolds numbers.

**Figure 11:**
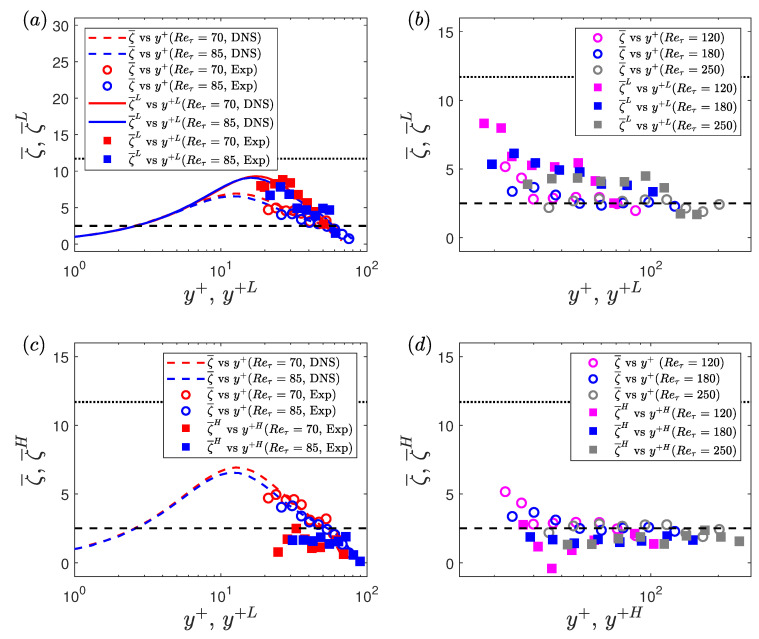
Unconditional (open circles) and conditionally averaged (closed squares) indicator functions for (**a**) $Re_{\tau }$ = 70 and 85, and for (**b**) $Re_{\tau }$ = 120, 180 and 250 during low-drag events. Unconditional (open circles) and conditionally averaged (closed squares) indicator functions for (**c**) $Re_{\tau }$ = 70 and 85, and for (**d**) $Re_{\tau }$ = 120, 180 and 250 during high-drag events. The criteria to detect a low-drag event is $\Delta t_{cr}^{+}=200$ and $\tau_{w}/\bar{\tau_{w}}<$ 0.9, and a high-drag event is $\Delta t_{cr}^{+}=200$ and $\tau_{w}/\bar{\tau_{w}}>$ 1.1. Dashed lines represent 2.5 and dotted lines represent 11.7.

To further investigate the slope of the conditional velocity profiles, the so-called indicator function is calculated, which is generally used to study the logarithmic dependence of the mean velocity profile [44]. For the unconditional velocity data, the indicator function is given by: $\bar{\zeta }=y^{+}d{\bar{U}}^{+}/dy^{+}$. For the conditional velocity data, the indicator functions are given by ${\bar{\zeta }}^{L}=y^{+L}d{\bar{U}}^{+L}/dy^{+L}$ and ${\bar{\zeta }}^{H}=y^{+H}d{\bar{U}}^{+H}/dy^{+H}$ for the low- and high-drag events, respectively. The profiles of indicator function are shown in Figure 11. It can be seen that that for $Re_{\tau }$ = 70 and 85, the $\bar{\zeta }$ profiles do not exhibit a logarithmic dependence. For $Re_{\tau }$ = 120, 180 and 250, the $\bar{\zeta }$ profiles approximately collapse on the value of 1/κ=2.5 for $y^{+}\geq 30$, thus suggesting a logarithmic dependence. Here, κ is the von Kármán constant. It is observed from Figure 11a,b that the ${\bar{\zeta }}^{L}$ profiles at all Reynolds numbers are closer to the Virk’s MDR (1/κ=11.7) for $y^{+}\leq 30$. For $Re_{\tau }$ = 120, 180 and 250, the ${\bar{\zeta }}^{L}$ profiles remain above the unconditional profiles for $y^{+}\geq 30$, thus showing that the slope of the low-drag velocity profiles is slightly higher than the unconditional profiles in the log-law region. Figure 11c,d shows that the ${\bar{\zeta }}^{H}$ profiles at $Re_{\tau }$ = 70 and 85, are lower than the $\bar{\zeta }$ profiles (except close to the centreline), with the effect being more significant for $y^{+}\leq 30$. For $Re_{\tau }$ = 120, 180 and 250, the slope of the ${\bar{\zeta }}^{H}$ profiles is slightly lower than the $\bar{\zeta }$ profiles for all wall-normal locations.

### 7.2. Similarity between Turbulent Drag Reduction and Low-Drag Events in Newtonian Turbulence

To quantify the “drag reduction” during the low-drag events a percentage decrease in the wall shear stress, during these low-drag events, is calculated. The comparison with the drag-reduction literature is carried out only for $Re_{\tau }$ = 180 and 250. It is found that the percentage drag reduction is about 36% for $Re_{\tau }$ = 180 and 250 when calculated using Equation (5).

(5) $$\%DR=\frac{\bar{\tau_{w}}-{\bar{\tau_{w}}}^{L}}{\bar{\tau_{w}}}\approx 36\%\left(Re_{\tau }=180\mathrm{and}250\right).$$

This level of drag reduction is similar to some of the other techniques employed previously to reduce drag in channel flows. For example, when using polymer additives at low concentration, the low-drag reduction (LDR) regime is observed [45,46]. A comparison is made with the experimental data obtained by Warholic et al. [45] at $Re_{h}\approx$ 20,000 for the case where a drag reduction of about 33% was observed. Drag reduction due to superhydrophobic surfaces were investigated by Min and Kim [47]. They conducted DNS in a channel flow for $Re_{\tau }$ = 180 (for DR = 0) and by using streamwise slip, they obtained a maximum drag reduction of 29%. Choi et al. [48] implemented DNS in a channel flow at $Re_{\tau }$ = 180 (for DR = 0) to numerically study the effect of blowing and suction on the skin-friction drag. They employed out-of-phase boundary conditions for the spanwise and wall-normal velocities to simulate the blowing and suction effects on the channel, and obtained a drag reduction of about 26% by applying spanwise control.

**Figure 12:**
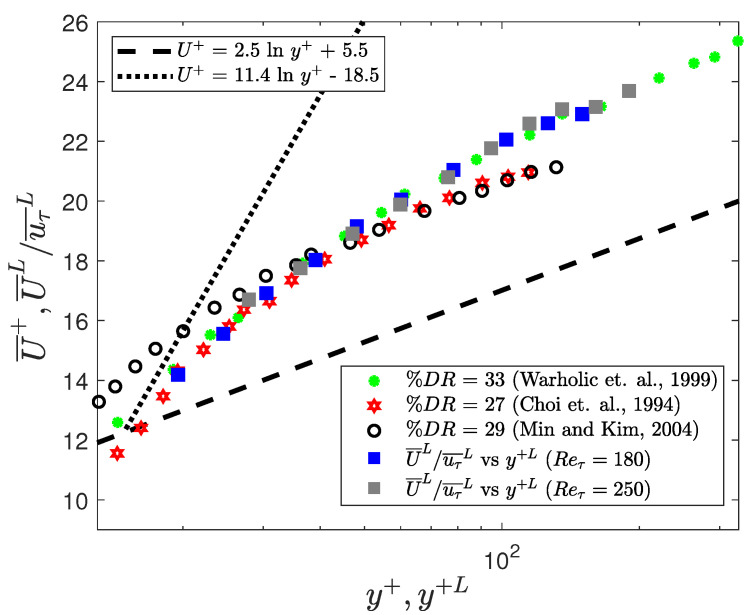
Conditional streamwise velocity profiles for $Re_{\tau }$ = 180 and 250 during the low-drag events. Streamwise velocity profiles, where different drag reduction mechanisms are employed previously: Warholic et al. [45] used polymeric additive, Min and Kim [47] used hydrophobic surface in the form of slip-boundary condition for the streamwise direction and Choi et al. [48] applied out-of-phase boundary condition to the spanwise velocity at the surface. Dashed line represents the Prandtl-von Kármán log-law: $U^{+}$ = 2.5 ln $y^{+}+5.5$ and dotted line represents the lower end of the 95% confidence interval of the Virk’s MDR asymptote: $U^{+}$ = 11.4 ln $y^{+}-18.5$ [43].

In Figure 12, a comparison is shown between the streamwise velocity profiles obtained using these three techniques for turbulent drag reduction and the conditional streamwise velocity profile obtained in the present experiment at $Re_{\tau }$ = 180 and 250.

A good agreement can be seen between the conditionally averaged profile for $Re_{\tau }$ = 180 and 250 and the profile obtained by Warholic et al. [45] for DR=33% using polymer additives. The profiles obtained by Min and Kim [47] and Choi et al. [48], and the present experiment are also in relatively good agreement with the obvious difference arising due to the lower levels of drag reduction reported in these cases. One major difference in the result obtained by Min and Kim [47] is that the velocity profile shifts upwards even closer to the wall which is the consequence of the slip boundary condition. Therefore, it suggests that for the fully-turbulent flows ($Re_{\tau }=$ 180 and 250), the conditional streamwise velocity for $y^{+}≳20$ during the low-drag events mimics the flow as observed during the LDR phenomenon due to polymer addition or the drag reduction due to spanwise oscillation. For the case of superhydrophobicity, this similarity between the velocity profiles can be observed approximately in the log-law region. Thus, if a method could be found to encourage the turbulent state to enter the low-drag “hibernating” state more often, a significant time-averaged drag reduction would be achievable.

### 7.3. Reynolds Shear Stress

DNS studies by Park and Graham [13] and Xi and Graham [12], using MFU at $Re_{\tau }=85$, showed that the Reynolds shear stress drops to a very low value during the low-drag events. There is still no information in the prior literature regarding the RSS characteristics, during the conditional events, from either physical experiments or from DNS in extended domains. For the experiments (discussed in Section 2), two-component (streamwise and wall-normal) velocity measurements have been made for $Re_{\tau }$ = 70 and 85 to study the behaviour of the Reynolds shear stress during the conditional events. To carry out the conditional sampling, each wall-normal location is sampled for 2 h while simultaneously measuring the wall shear stress using HFA. DNS study is conducted for $Re_{\tau }$ = 70 and 85 which provides the streamwise and wall-normal velocity information for various wall-normal locations (discussed in Section 3).

**Figure 13:**
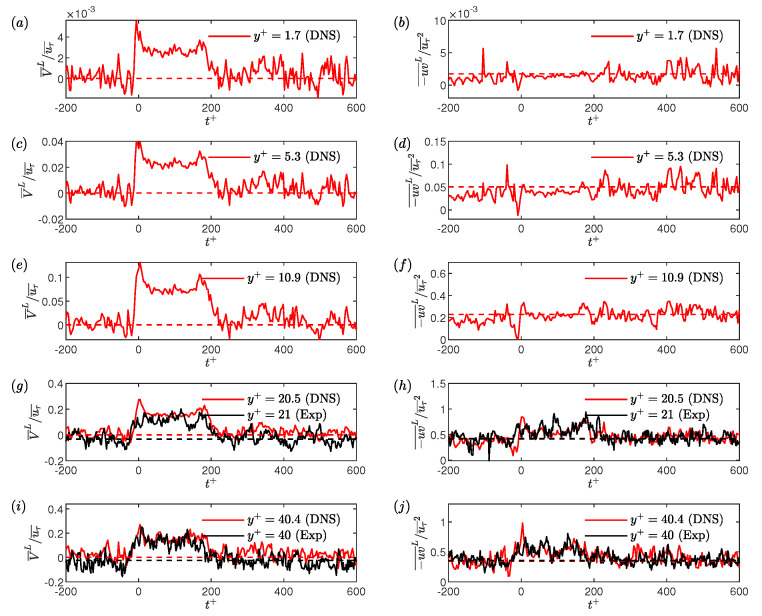
Ensemble-averaged wall-normal velocities (**a**,**c**,**e**,**g**,**i**) and Reynolds shear stresses (**b**,**d**,**f**,**h**,**j**) obtained using DNS (red solid lines) and experiment (black solid lines) during low-drag events for $Re_{\tau }$ = 70. Here, $t^{+}=0$ indicates start of low-drag events. The time-averaged values for the corresponding wall-normal locations are shown using red dashed lines (obtained using DNS) and black dashed lines (obtained using experiment). The criteria to detect a low-drag event is $\Delta t_{cr}^{+}=200$ and $\tau_{w}/\bar{\tau_{w}}<$ 0.9.

To calculate the conditional RSS, the streamwise velocity fluctuations and the wall-normal velocity fluctuations during the conditional events are calculated by subtracting their time-averaged values from the instantaneous conditional values. Figure 13 shows the ensemble averaged wall-normal velocities (${\bar{V}}^{L}$) and ensemble averaged Reynolds shear stress ($-{\bar{uv}}^{L}$). All the quantities are normalised by the time-averaged friction velocity ($\bar{u_{\tau }}$). The threshold and time-duration criteria to detect a low-drag events are $\tau_{w}/\bar{\tau_{w}}<$ 0.9 and $\Delta t_{cr}^{+}=200$, respectively. For $y^{+}<21$, experimental data are not available and therefore only DNS results are shown. A fairly good agreement between the experimentally and numerically obtained ensemble-averaged wall-normal velocity and RSS is observed. From continuity, the time-averaged wall-normal velocity must be zero, as can be observed from the DNS data. There is a slight discrepancy in the time-averaged values for the experimental data which is attributed to the error associated with the LDV measurements (discussed in Section 2). The conditionally averaged wall-normal velocity is higher than the time-averaged value during the low-drag events.

**Figure 14:**
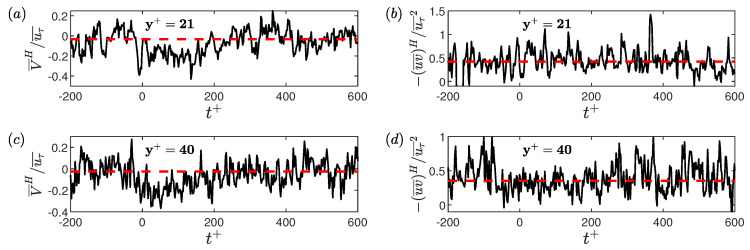
Ensemble-averaged wall-normal velocities (**a**,**c**) and Reynolds shear stresses (**b**,**d**) obtained using experiment during high-drag events for $Re_{\tau }$ = 70. Here, $t^{+}=0$ indicates start of high-drag events. The time-averaged values for the corresponding wall-normal locations are shown using red dashed lines (obtained using experiment). The criteria to detect a high-drag event is $\Delta t_{cr}^{+}=200$ and $\tau_{w}/\bar{\tau_{w}}>$ 1.1.

The ensemble averaged streamwise velocities have already been shown previously in Section 7.1. Based on the conditionally-averaged streamwise and wall-normal velocities, it can be said that the low-drag events form a subset of so-called Q2 events, i.e., u<0 and v>0. Figure 14 shows the ensemble-averaged wall-normal velocity and RSS during the high-drag events for $y^{+}=$ 21 and 40. The ensemble averaged wall-normal velocity is lower than the time-averaged wall-normal velocity whereas the ensemble averaged RSS is unchanged. Again, based on the conditionally-averaged streamwise and wall-normal velocities, it can be said that the high-drag events form a subset of Q4 events, i.e., u>0 and v<0. This behaviour will be further investigated in the following discussions.

**Figure 15:**
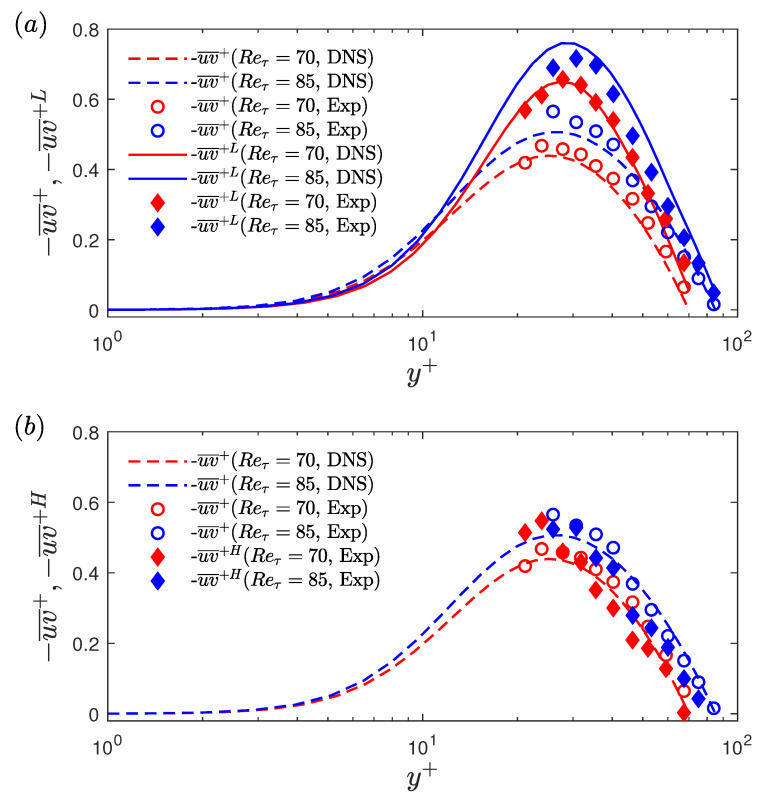
(**a**) Unconditional and conditionally averaged RSS profiles for $Re_{\tau }$ = 70 and 85 during low-drag events. (**b**) Unconditional and conditionally averaged RSS profiles for $Re_{\tau }$ = 70 and 85 during high-drag events.

The unconditional and conditionally-averaged RSS profiles, obtained for these two Reynolds numbers, shown in Figure 15. A good agreement can be observed between the experimental and DNS unconditional profiles. The conditionally-averaged data are normalised using ${\bar{u_{\tau }}}^{2}$, are shown in Figure 15a,b, for low- and high-drag events, respectively. For the low-drag case, both experimental and DNS results are shown, and for high-drag case only experimental results are shown. A good agreement is observed between the conditionally averaged profiles obtained using experiments and DNS, with a slight discrepancy observed for the $Re_{\tau }=85$ results. As seen in Figure 15a, the conditionally averaged profiles have slightly lower values than the unconditional profiles for $y^{+}≲10$. For $y^{+}≳10$ the conditionally averaged profiles are higher than the unconditional profiles with the effect being more significant for $y^{+}$ between 20 and 40. For the high-drag case, as seen in Figure 15b, the conditionally-averaged RSS profiles almost collapse onto the unconditional profiles for all the wall-normal locations measured. This result suggests that the Reynolds shear stress is more affected by the low-drag events compared to the high-drag events. A sensitivity check has been executed to study the effect of changing the criteria for conditional events on the conditional RSS profiles for $Re_{\tau }$ = 70. No significant dependence of the RSS profiles is observed for the different values of criteria studied here.

**Figure 16:**
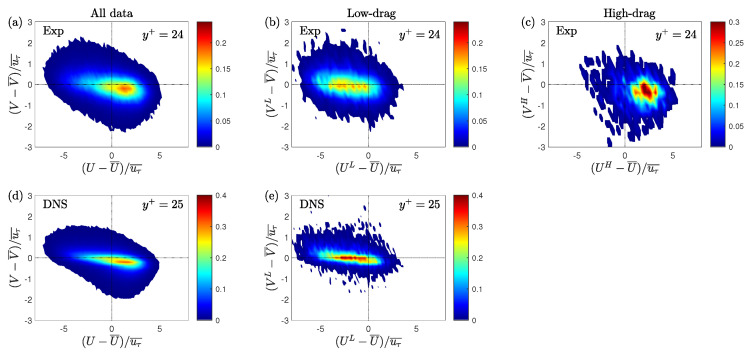
Unconditional (**a**), low-drag (**b**) and high-drag (**c**) jpdfs of streamwise and wall-normal velocity fluctuations for $y^{+}=24$ at $Re_{\tau }$ = 70 using experiments. Unconditional (**d**) and low-drag (**e**) jpdfs of streamwise and wall-normal velocity fluctuations for $y^{+}=25$ at $Re_{\tau }$ = 70 using DNS. Unconditional and conditional velocity fluctuations are normalised using the time-averaged $\bar{u_{\tau }}$.

A quadrant analysis is conducted to calculate the contribution to the Reynolds shear stress from various turbulent events [49]. In quadrant analysis, the Reynolds shear stress is divided into four quadrants based on the signs of the streamwise and wall-normal velocity fluctuations: Q1 (+u, +v), Q2 (−u, +v), Q3 (−u, −v) and Q4 (+u, −v). The Q2 and Q4 events are generally related to the ejection and sweep events, respectively [49]. Here, the normalisation of both unconditional and conditional velocity fluctuations is based on the time-averaged friction velocity ($\bar{u_{\tau }}$). For unconditional velocity fluctuations, the time-averaged velocities are subtracted from the instantaneous velocities, and for the conditional velocity fluctuations, the time-averaged velocities are subtracted from the instantaneous conditional velocities during the low- or high-drag events. Figure 16a,d shows the jpdfs (joint probability density functions) of the unconditional streamwise and wall-normal velocity fluctuations for $Re_{\tau }$ = 70 obtained using the experiment (at $y^{+}$ = 24) and DNS (at $y^{+}$ = 25). The shape of the unconditional jpdfs are roughly elliptical with their major axes tilted in the direction of Q2 and Q4 motions. During the low-drag events the jpdf shifts towards the Q2 quadrant, whereas during the high-drag events the jpdf shifts towards the Q4 quadrant.

This observation is consistent with the previous results where it is shown that during the low-drag events the ensemble-averaged streamwise decreases and wall-normal velocities increases for $y^{+}\approx$ 20–40, whereas the opposite is true for high-drag events.

**Figure 17:**
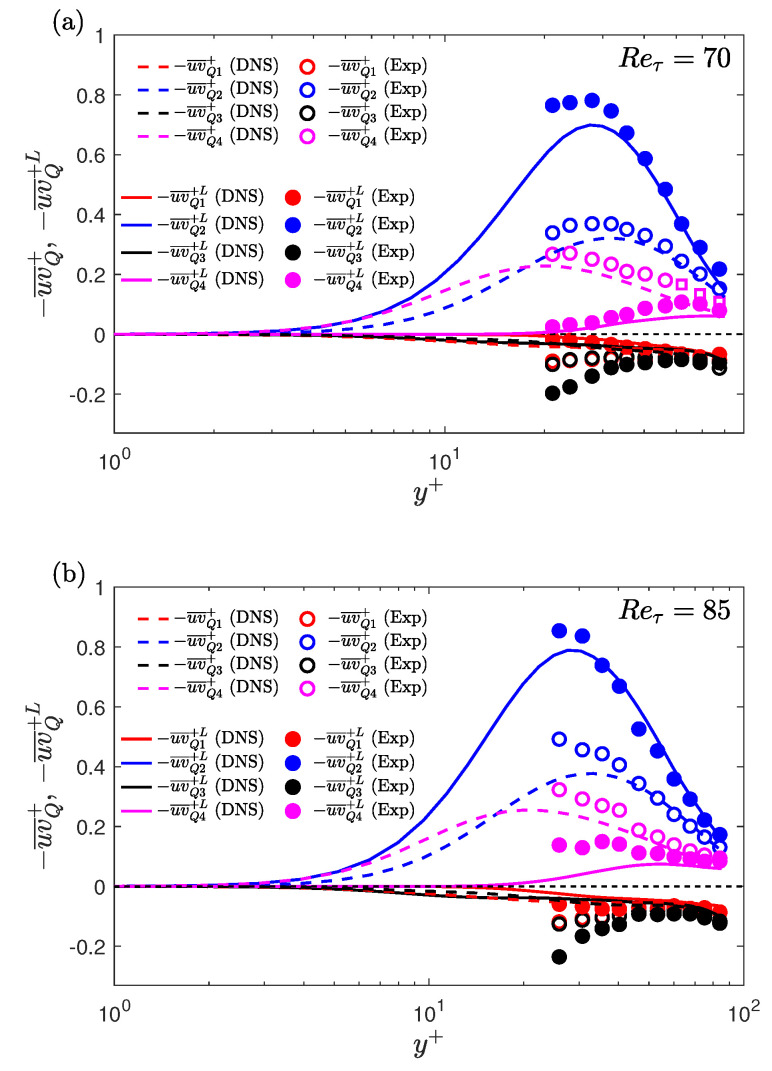
(**a**) Contribution to $-\bar{uv}$ from different quadrants for the unconditional case and during the low-drag events for (**a**) $Re_{\tau }$ = 70 and (**b**) $Re_{\tau }$ = 85. The criteria to detect a low-drag event is $\Delta t_{cr}^{+}=200$ and $\tau_{w}/\bar{\tau_{w}}<$ 0.9. Thin black dashed line represents a constant value of zero.

Figure 17a,b shows the unconditional and conditional (low-drag) profiles of contribution from the various quadrants in the Reynolds shear stress for $Re_{\tau }$ = 70 and $Re_{\tau }$ = 85, respectively. Figure 16b,d shows the joint distribution of streamwise and wall-normal velocity fluctuations during the low-drag events for $Re_{\tau }$ = 70, obtained using experiments (at $y^{+}$ = 24) and DNS (at $y^{+}$ = 25), respectively. Figure 16c shows the joint distribution during the high-drag events for $Re_{\tau }$ = 70 at $y^{+}$ = 24, obtained using experiments. A good qualitative agreement is observed between the experimental and DNS results for the unconditional data. It can be seen that the major contributors to the Reynolds shear stress are the Q2 and Q4 motions, which explains the reason for the tilted shape of the jpdf shown in Figure 16a,d. These two quadrants are considered to be responsible for the turbulence production [50,51]. It is also observed that the Q4 motions or the “sweep” type motions are the most dominant motions for $y^{+}≲20$ and for the higher wall-normal locations Q2 motions or the “ejection” type motions are the most dominant. For the low-drag case, the Q2 events contribute more than the other quadrants for all the wall-normal locations at both $Re_{\tau }$ = 70 and 85. Another interesting observation is that the Q4 events contribution decreases to a very low value during these low-drag events. This further reinforces the hypothesis that the low-drag events are composed of low-streamwise speed and upwash motions. There is a good qualitative and also fairly good quantitative (for $y^{+}≳$ 30–40) agreement between the experimental and DNS results. The discrepancies between the experimental and DNS data in the conditional data are aligned with their unconditional values, which suggests that these slight variations are the result of noise in the measurement rather than different physical observations.

The observation from the quadrant contributions is consistent with the previous numerical findings by Kushwaha et al. [22] where it is shown that the low-wall shear stress events are associated with counter-rotating streamwise vortex pairs transferring momentum away from the wall. Park et al. [16] showed in MFU simulations that the low-drag event is the precursor to a strong bursting event which is again consistent with the present result. The low-speed fluid moves away from the wall (ejection process) during these low-drag events which ultimately undergo a bursting process. The ejection and bursting processes are well studied in the past in regards to the low-speed streaks moving away from the wall and bursting in the buffer layer region (for more details, see in [52,53]). Adrian et al. [54] provided a hairpin vortex model in an effort to unify the various previous findings related to the coherent structures observed in the turbulent boundary layer. It was stated that the hairpin vortex originates from the wall inducing a region of low speed between two legs of the vortex which then lifts up by ejection process. The present work suggests that the low wall shear stress events are representative of low-speed regions which are generally observed between the legs of the hairpin vortices in wall-bounded turbulent flows [54,55]. Although it should be noted that the present work employs a different criterion to detect these low-drag events ($\tau_{w}/\bar{\tau_{w}}<0.9$ and $\Delta t^{+}>200$) and therefore these conditional events form only a subset of the low-speed streaks/events observed in the past [55].

**Figure 18:**
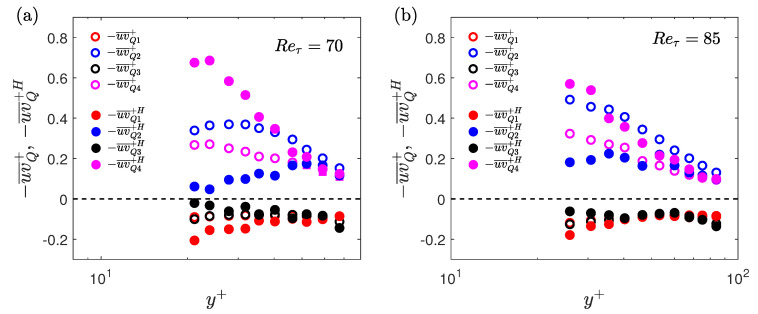
(**a**) Contribution to $-\bar{uv}$ from different quadrants for the unconditional case and during the high-drag events for (**a**) $Re_{\tau }$ = 70 and (**b**) $Re_{\tau }$ = 85. The criteria to detect a high-drag event is $\Delta t_{cr}^{+}=200$ and $\tau_{w}/\bar{\tau_{w}}<$ 0.9. Thin black dashed line represents a constant value of zero.

Results for the high-drag events are shown in Figure 18a,b for $Re_{\tau }$ = 70 and 85, respectively. It can be observed that during the high-drag events, the Q4 events are the significant contributor to the Reynolds shear stress. This is again expected based on the ensemble-averaged data, i.e., high-drag events are composed of high-speed and downwash motions for $y^{+}\geq 20$.

## 8. Summary

An investigation into the intermittencies associated with the low- and high-drag events in turbulent channel flow has been conducted using experiments and DNS. For experiments, simultaneous measurements of streamwise velocity and wall shear stress are carried out to detect and characterise these intermittencies for $Re_{\tau }$ between 70 and 250. DNS is carried out in a large computational box for $Re_{\tau }$ = 70 and 85. The fraction of time spent in the intervals of low- and high-drag is found to be roughly independent of the Reynolds number for $70\leq Re_{\tau }\leq 250$ when the criteria for minimum time-duration is kept constant in inner units. The low- and high-drag events exhibit an exponential distribution of the frequency of their occurrence when studied as a function of the duration of their intervals. It is found that even for artificially constructed signals (up to the limit of Gaussian signal), there is a presence of spikes and dips in the ensemble-averaged data, if the same criteria is applied as used to detect a low- or high-drag event in the wall shear stress signals. This suggests that these spikes (or dips) might be the consequence of the conditional averaging of a time series data.

Streamwise velocity profiles, conditionally sampled during the low-drag events, get closer to Virk’s MDR profile and the lower-branch of the nonlinear TW solutions for $y^{+}\approx$ 20–35 at all studied Reynolds numbers. For $120\leq Re_{\tau }\leq 250$, in the log-law region, the conditional velocity profile is higher than the unconditional velocity profile with the slope of the profile higher during the low-drag events. Similarly, the conditional velocity profile is lower than the unconditional velocity profile with the slope of the profile being slightly lower during the high-drag events. A comparison of the conditional streamwise velocity profiles at $Re_{\tau }$ = 180 and 250 with other drag reduction techniques is made. A good agreement between the profiles in the log-law region is observed. For $Re_{\tau }$ = 70 and 85, in addition to the streamwise velocity, wall-normal velocity is also measured to investigate the behaviour of RSS. There is found to be an increase in the conditionally averaged RSS for $y^{+}≳10$ during the low-drag events. This is observed to be due to a significant increase in the turbulence-generating Q2 motions during these low-drag events. The high-drag events are found to be associated with the Q4 events, although the RSS during these events remain fairly similar to the unconditional profile for $y^{+}≳20$.

## Figures and Tables

**Table 1:** Reynolds numbers and various wall-normal locations studied. Parameters measured for each Reynolds numbers are also shown.

Reτ	$y^{+}$	Parameters
70	21, 24, 28, 32, 35, 40, 46, 51, 60, 68	$\tau_{w},U,V$
85	26, 31, 36, 32, 41, 47, 54, 61, 76, 85	$\tau_{w},U,V$
120	22, 26, 30, 37, 46, 59, 71, 85, 103	$\tau_{w},U$
180	24, 30, 38, 48, 60, 75, 98, 128, 157	$\tau_{w},U$
250	35, 45, 58, 74, 94, 118, 143, 171, 202, 242	$\tau_{w},U$

**Table 2:** Rate of decay (λ) of the PDF of occurrence of conditional events for $100\leq \Delta t^{+}\leq 300$ at various Reynolds numbers. Numbers in brackets correspond to the $R^{2}$ value. The threshold criteria for low- and high-drag events are $\tau /\bar{\tau_{w}}<0.9$ and $\tau /\bar{\tau_{w}}>1.1$, respectively.

Reτ	Low-Drag	High-Drag
70	0.0192 (0.89)	0.0258 (0.90)
85	0.0181 (0.95)	0.0252 (0.97)
120	0.0185 (0.98)	0.0245 (0.97)
180	0.0185 (0.96)	0.0251 (0.97)
250	0.0183 (0.99)	0.0245 (0.99)
